# The Rage Attack Questionnaire-Revised (RAQ-R): Assessing Rage Attacks in Adults With Tourette Syndrome

**DOI:** 10.3389/fpsyt.2019.00956

**Published:** 2020-01-28

**Authors:** Kirsten R. Müller-Vahl, Lena Kayser, Anna Pisarenko, Martina Haas, Nikolas Psathakis, Lisa Palm, Ewgeni Jakubovski

**Affiliations:** Clinic of Psychiatry, Social Psychiatry and Psychotherapy, Hannover Medical School, Hannover, Germany

**Keywords:** rage attacks, rage attack questionnaire-revised, tourette syndrome, tics, attention deficit/hyperactivity disorder, outbursts, adults, questionnaire

## Abstract

**Introduction:**

Although defined by the presence of tics, most patients with Gilles de la Tourette syndrome (TS) also suffer from different psychiatric disorders. While much is known about clinical characteristics of comorbidities such as attention deficit/hyperactivity disorder (ADHD), obsessive compulsive disorder (OCD), depression, and anxiety disorders, only very little is known about rage attacks. Most of this data is based on small studies in children. Until today no larger studies have been performed in adults with TS—most likely because of the lack of validated instruments. The aim of this study was to develop a new assessment and investigate rage attacks in a large sample of adults with TS and healthy individuals.

**Materials and Methods:**

Based on a parent questionnaire for children with TS, we generated 27 items for a revised version of a rage attack questionnaire (RAQ-R) and tested factor structure, internal consistency, as well as convergent and discriminant validity. We used an online survey and included 127 patients with TS and 645 control subjects. In addition to the RAQ-R, we used several other self-assessments to measure tic severity, quality of life, as well as several psychiatric symptoms including ADHD, OCD, depression, anxiety, and impulsivity.

**Results:**

Based on expert option and statistical analyses [including item-total correlation, skewness, inter-item correlation, and principal component analysis (PCA)], we performed an item reduction resulting in a final, 22-items version of the RAQ-R (range, 0–66). Investigating internal consistency, discriminant validity, test reliability, and factor structure, the RAQ-R demonstrated good to excellent quality criteria. As assessed by RAQ-R, rage attacks were significantly more common in patients with TS compared to controls (p < 0.001). Rage attacks could be clearly differentiated from the phenomenon of impulsivity. Although rage attacks occurred more often in individuals with ADHD, they were also found in patients with “TS only”, independently from comorbid ADHD, impulsivity, and OCD. Rage attacks were found to significantly influence patients’ quality of life.

**Conclusions:**

Thus, from our data based on a large sample it is suggested that rage attacks represent a discrete comorbidity in adults with TS.

## Introduction

For many years it is well-known that most of the patients with Gilles de la Tourette syndrome (TS) suffer not only from motor and vocal tics, but also from a large spectrum of psychiatric disorders. The most common and best studied comorbidities are attention deficit/hyperactivity disorder (ADHD) and obsessive compulsive disorder (OCD). However, other psychiatric symptoms may occur as well such as depression, anxiety disorders, sleep disorders, personality disorders, and self-injurious behavior. Some further conditions are mainly described in children population such as learning disabilities, autism spectrum disorders, and rage attacks (1).

Although clinically often reported by patients and their families, until today, only very little is known about the phenomenon of episodic rage attacks in patients with TS. This is even more surprising since—according to current literature—rage attacks occur in 25 to 70% of patients (2, 3) and are considered the most important problem for the families (4). Most typically, rage attacks appear suddenly and without any warning “out of nowhere”. They are unpredictable and unrelated to any environmental trigger. Although longitudinal studies are lacking, from clinical experience it seems that rage attacks do not improve with age (1). Thus, because of the assumed high prevalence rate, the persistence into adulthood, the unpredictable nature, and the inability to interrupt, once the episode has started, rage attacks significantly impair not only patients’ quality of life, but also family life and social relationships. In line with this data, already in 1985, Comings and Comings (5) stated: “If there is a single word that best characterized the behavioral problems in TS it would be *anger*”.

A major problem with respect to clinical research is that there is not only no clear definition for rage attacks in TS, but also no validated assessment. Even the terminology used is inconsistent and in the literature several different terms have been suggested to describe more or less the same phenomenon including emotion control difficulties or emotional dysregulation (6), disinhibited behavior (1), anger control problems (3), explosive outbursts (2), rage outbursts (6), episodic rage or episodic rage attacks (7), and aggression or aggressive behavior (8).

From available small studies—most of them performed in children and adolescents—it is suggested that rage attacks are closely related to tic severity and the presence of comorbid disorders, in particular, comorbid ADHD, OCD, oppositional defiant disorder (ODD), and impulsivity, but do not occur in patients with “TS only” (without comorbidities) (2, 9–13). Accordingly it is believed that rage attacks do not represent a discrete disorder—comparable to depression and anxiety—but should be interpreted as an expression of a disinhibited behavior (1, 9).

In recent studies, different assessments have been used to assess rage attacks in patients with TS. For example, Stephens and Sandor (8) used the aggression subscales of the Achenbach’s Child Behavior Checklist (CBCL) completed by parents, the Teacher’s Report Form (TRF), and the conduct disorder subscale of the Conner’s Parent Rating Scale (CPRS) to investigate “aggressive behavior” in children with TS (n = 33, age range, 6–14 years) compared to healthy controls (n = 6, age range, 7–12 years). They found that aggressive behavior was independent from tic severity, but associated with comorbid ADHD and OCD, while children with “TS only” did not differ from healthy controls. Cavanna et al. (14) assessed “anger” in 25 adolescents and young adults with TS (mean age = 15.4 ± 2.6 years, range, 12.2–22.4 years) compared to 41 healthy controls (mean age = 16.3 ± 2.9 years, range, 13.0–22.8 years) using the State Trait Anger Expression Inventory (STAXI), the CBCL (completed by parents and teachers) and the Conner’s Parent (CPRS-R) and Teacher Rating Scales-Revised (CTRS-R). While STAXI scores failed to demonstrate any significant differences between patients and controls, most subscores of the CBCL, CPRS-R, and CTRS-R were significantly higher in the TS group. The authors therefore concluded that anger expression represents a clinically relevant symptom in patients with TS, but measures such as the STAXI might be not sensitive enough to assess this symptom adequately. Most importantly and in contrast to most other reports (7, 8, 10), in this study aggression symptoms did not occur more common in patients with “TS+ADHD” compared to those with “TS-ADHD”. Using the questionnaire for Intermittent Explosive Disorder (IED) according to DSM-IV, Chen et al. (10) investigated 218 patients with TS (mean age = 17.8 ± 12.8 years) and found “explosive outbursts” in 20% of the patients. Several clinical characteristics were strongly associated with explosive outbursts including comorbid ADHD and tic severity.

To measure rage attacks more specifically, Budman et al. (9) developed a “Rage Attacks Questionnaire” including 22 items. Based on data in 48 children with TS (mean age = 11.4 ± 2.9 years), cluster solution revealed four homogeneous clinical subgroups: (i) specific urge resolution, (ii) environmentally secure reactivity, (iii) nonspecific urge resolution, and (iv) labile nonresolving. The authors concluded that episodic rage should not be considered an independent disorder, but viewed as a nonspecific symptom that may result from a wide range of psychological, biological, and environmental conditions. However, this study has several limitations: (i) inclusion of only a small sample size, (ii) possible influence of medication, (iii) inclusion of only children, and (iv) inclusion of only patients with pre-diagnosed comorbid rage attacks. In addition, the assessment itself—although more specific than previously used rating scales—is limited by the inclusion of open-ended and closed-ended questions, lack of measurements of reliability and validity, and the fact that it was developed for children only completed by their parents.

This study was designed to overcome these limitations of recent studies. Therefore, we developed and validated a revised version of a “rage attack questionnaire” (RAQ-R) in a large sample of adult patients with TS and healthy controls. For this purpose, we used a principal component analysis (PCA) and tested internal consistency as well as convergent and discriminant validity. Since specific instruments are missing to assess rage attacks in adults, for convergent validity we used measurements for impulsivity, because it has been suggested that these symptoms are closely related (7).

## Materials and Methods

### Item Generation of the Rage Attack Questionnaire-Revised (RAQ-R)

Following the definition given by Budman et al. (9) rage attacks were defined as follows:

“Rage attacks are sudden, mostly short-lived, intensive, impulsive, emotional reactions to situations and/or stimuli that cannot be controlled. The behavior is totally out of proportion to the trigger event. Rage attacks may manifest in inappropriate verbal utterances, property damage, or aggressive actions. Those affected are aware of the disproportionate nature of their behavior. Therefore, rage attacks are perceived as unpleasant, unintentional, and often shame-filled. Nonetheless, affected persons feel unable to change their behavior.”

According to this definition and based on our and other experts experience, we believed that rage attacks represent a clinically different phenomenon compared to anger as assessed by STAXI and other anger-related assessments. We hypothesized that rage attacks occur more often and intense in patients with TS, but represent a phenomenon of “normal behavior” and therefore also occur—in milder form—in healthy people.

The items for the RAQ-R were inspired by items of the “rage attacks questionnaire” developed by Budman et al. (9). However, in contrast to Budman’s questionnaire, the RAQ-R was developed as a self-assessment for adults with TS. In addition, we decided to include only closed-ended questions so that the RAQ-R can be used to assess intensity and frequency of rage attacks.

The RAQ-R was generated by an interdisciplinary team of experts with extensive clinical experience in patients with TS. Based on above described criteria, nine items of the RAQ-R resemble to Budman’s questionnaire (9). Based on clinical experience, 18 further questions were added to cover the whole spectrum of the phenomenon of rage attacks including all forms and severities. After revision by members of our research group, TS experts from other specialty clinics were asked for revision. Thereafter, five patients with TS were asked to complete the RAQ-R. None of them reported any problems. Thus, the initial version of the RAQ-R contained 27 items, all of which were scaled from 0 to 3 on a Likert scale. The item scores were labeled: 0 = not at all/never, 1 = a little/sometimes, 2 = strong/frequent, and 3 = very strong/very common. The total score is obtained by summing all items [see RAQ-R (German and English 22-items versions) and paragraph: *Final version of the RAQ-R* [shows items that have been excluded)].

### Inclusion Criteria

We included patients with TS and other primary chronic tic disorders (chronic motor and vocal tic disorder)—subsequently, the abbreviation TS is used for all patients with tic disorders—and healthy controls. Inclusion criteria were: (i) age≥18 years, (ii) internet access, and (iii) knowledge of German language. Patients were recruited from our Tourette outpatient clinic and *via* German advocacy groups. This restricted recruitment strategy was used to ensure participation of only patients diagnosed with TS. Healthy controls were recruited *via* intranet among staff and students at Hannover Medical School (MHH).

### Study Design and Assessments

The study was carried out online *via* the SoSci Survey platform (without any personal investigation). Accordingly, only self-assessments could be used. Each participant received a direct link to access the online platform. Besides the RAQ-R (27-items version), the survey consisted of questions on demographic data (age: for reasons of data protection clustered in groups: 1 = 18–25 years, 2 = 26–35 years, 3 = 36–45 years, 4 = 46–55 years, 5 = 56–65 years, and 6 > 65 years, gender, level of education, previous psychiatric diagnoses, current medication), and the following instruments to assess a broad spectrum of further psychiatric symptoms as well as tic severity and quality of life:

- Barratt Impulsivness Scale—Short Version (BIS-15) (15): to assess impulsivity. It consists of three subscales (non-planning, motor, and attentional impulsivity). The BIS-15 has been shown to be very economical and have good psychometric properties.
- Impulsive behavior scale-8 (I-8) (16): to measure impulsivity. This short, reliable, and valid method includes four subscales (urgency, lack of intention, lack of perseverance, and risk-taking).
- ADHD self-assessment scale (ADHD-SB) (17): a screening method for ADHD. It was found to have good internal consistency, test-retest reliability, convergent, and discriminant validity.
- ICD-10 Symptom Rating (ISR) (18): a tool for screening for mental illnesses in adolescents and adults including the subscales: depressive syndrome, anxiety syndrome, obsessive-compulsive syndrome, somatoform syndrome, eating disorder syndrome, and supplementary items. Previous studies indicated satisfactory to good psychometric properties.
- Obsessive-Compulsive Inventory-Revised (OCI-R) (19): to measure obsessive-compulsive symptoms in six subscales (washing, controlling, organizing, obsessive thoughts, hoarding, and neutralizing). The psychometric properties were shown to be very good.
- Adult Tic Questionnaire (ATQ) (20): a self-assessment to measure tic severity with good psychometric properties.
- Gilles de la Tourette Syndrome—Quality of Life Scale (GTS-QoL) (21): to measure specifically the impact of TS on quality of life with four subscales (psychological, physical, obsessional, and cognitive).
- Visual analogue scale (VAS) of the GTS-QoL (21): to assess the individuals’ current life satisfaction. The GTS-QoL and the VAS were been shown to have good psychometric properties.

All instruments, but the ATQ and the GTS-QoL, were used in both (healthy and TS) groups.

### Statistical Evaluation

#### Item Reduction of the RAQ-R

The initial version of the RAQ-R consisting of 27 items was examined for its psychometric properties in both samples separately. For each item we looked at the item-total correlation, skewness, inter-item correlations, as well as results obtained from the PCA.

#### Internal Consistency, Convergent, and Discriminant Validity

The factor structure of the final version of the RAQ-R was examined with a PCA. Internal consistency (reliability) was established *via* Cronbach’s alpha. Convergent and discriminant validity were tested *via* Pearson correlations of the RAQ-R with the impulsivity scales BIS-15 und I-8 (for convergent validity) and the ISR for discriminant validity. The psychometric testing was carried out using data of the control group. This was done in order to avoid distortions that could be caused by a particularly high amount of severe rage attacks in the TS group. The data from the TS group was used to establish correlations of the RAQ-R with TS specific questionnaires (ATQ and GTS-QoL) and for item selection.

#### Group Comparisons

A number of group comparisons *via* independent samples t-test were carried out to establish differences between the TS and the control group. The following variables were tested for group differences: age groups, gender, level of educational, and medication. For significant results Cohen’s d (22) as indicator of effect size was examined.

#### Missing Data

In case of missing data list-wise deletion was performed.

## Results

### Clinical Characteristics

A total of 772 subjects participated in this study: 127 subjects with TS and 645 control subjects. In the TS group, 115 individuals indicated that they have been diagnosed with TS, while 12 reported suffering from another chronic tic disorder. Altogether, 123 patients in the TS group rated their disease as being currently significant. For reasons of data protection, age was clustered in groups. On average the TS group was slightly older compared to the control group (CG), but the difference was not statistically significant [mean age groups (range, 1–6): TS: 2.74, CG: 2.57, p = 0.181]. As expected, there was a significant gender difference (p < 0.001): TS: 70.1% male (n = 89), 29.9% female (n = 38), CG: 20.6% male (n = 133), 79.4% female (n = 512). In general, the education level was relatively high and in the CG significantly higher (p < 0.001) than in the TS group: university degree: CG: 38.6% (n = 249), TS: 27.6% (n = 35), general qualification for university entrance: CG: 42.6% (n = 275), TS: 29.1% (n = 37), a general certificate of secondary education: CG: 17.4% (n = 112), TS: 28.3% (n = 36), and a certificate of secondary education: CG: 1.4% (n = 9), TS: 12.6% (n = 16). In the TS group, 2.4% (n = 3), but none in the CG, had not school degree.

As expected, the incidence of all pre-diagnosed psychiatric disorders (based on participants’ reports) was significantly higher in the TS group compared to the CG (for details see **Table 1**). Accordingly, 47.2% (n = 60) in the TS group, but only 2.3% (n = 15) in the CG reported being on medication for mental health problems or tics (p < 0.001). In line with this data, quality of life (according to QoL-VAS) was significantly lower in patients with TS compared to controls (TS: 7.08, CG: 8.76; p < 0.001).

**Table 1:** Frequency of different psychiatric diagnoses in each sample and current significance (according to patient information, not based on clinical assessments).

Diagnosis	Frequency TS (diagnosis currently of relevance) [n/%]	Frequency CG (diagnosis currently of relevance) [n/%]	Difference frequency (p)
OCD	53/42% (44/35%)	2/0% (1/0%)	<0.001
ADD	31/24% (24/19%)	4/1% (2/0%)	<0.001
Hyperactivity disorder	17/13% (9/7%)	1/0% (1/0%)	<0.001
ADHD	30/24% (23/18%)	3/0.5% (2/0%)	<0.001
Impulse control disorder	21/16.5% (18/14%)	1/0% (1/0%)	<0.001
Anxiety disorder	32/25% (24/19%)	13/2% (4/0.5%)	<0.001
Depression	53/42% (34/27%)	35/5% (18/3%)	<0.001
Insomnia	32/25% (31/24.5%)	18/3% (13/2%)	<0.001
Eating disorder	18/14 (11/9%)	17/3% (7/1%)	<0.001
Alcohol/drug addiction	5/4% (5/4%)	2/0% (0/0%)	0.04
Personality disorder	10/8% (7/5.5%)	3/0.5% (2/0%)	0.003
TS	115/91% (112/88%)	1/0% (1/0%)	<0.001
Other tic disorder	12/9% (11/9%)	0/0% (0/0%)	<0.001

OCD; obsessive-compulsive disorder, ADD; attention deficit disorder, ADHD; attention deficit/hyperactivity disorder, TS; Tourette syndrome, CG; control group.

### Item Reduction

To account for the demographical difference between the TS group and the CG, the item analysis was carried out separately in both samples. Based on clinical experience the following items were considered as highly clinically relevant and indispensable: 1, 4, 5, 7, 9, 10, 11, 15, 19, 20, 22, 23, 24, 25, and 27 (numbering of the items here and subsequently refers to the initial 27-items version of the RAQ-R, not the final 22-items version).

#### Item-Total Correlation

In both samples, the items 12 (CG: r_it_ = 0.15, TS: r_it_ = 0.32), 26 (CG: r_it_ = 0.342, TS: 0.34), and 27 (CG: r_it_ = 0.24, TS: r_it_ = 0.26) fell below or just above the minimally accepted correlation of r_it_ = 0.3 as recommended by Lienert and Raatz (23).

#### Skewness

The skewness indicates the type and strength of the asymmetry of a distribution of values. According to Fabrigar et al. (24) skewness should not exceed the value of 2. In the CG, items 6, 11, 12, 14, 16, 17, 20, 23, 24, 26, and 27 showed skewness values >2.0 and in the TS group, items 12 and 17. In addition in the TS group item 2 had a negative value indicating also a skewed distribution.

#### Inter-Item Correlation

A high correlation between two items indicates a possible redundancy, which can be lowered by eliminating one of these items. In the CG, the following items showed very high correlations (r > 0.7): 3 and 4 (r = 0.74) as well as 16 and 23 (r = 0.88). In the TS group, very high correlations were seen for the items 3 and 4 (r = 0.71), 3 and 15 (r = 0.71), 4 and 15 (r = 0.72), 7 and 9 (r = 0.70), 13 and 22 (r = 0.72), as well as 16 and 23 (r = 0.74) (for details see **Table 2**).

**Table 2:** Statistical item characteristics of the RAQ-R (data obtained from the control group, N = 645).

RAQ item	Mean	Standard deviation	Skewness	Corrected item-total correlation
1	.56	.567	.426	.671
2	1.02	.687	.347	.602
3	.64	.900	1.250	.765
4	.64	.730	1.046	.655
5	.19	.529	3.195	.615
6	.67	.766	1.006	.708
7	.57	.756	1.207	.739
8	.63	.754	1.057	.667
9	.69	.777	1.071	.524
10	.21	.532	2.877	.664
11	.34	.614	1.894	.667
12	.16	.439	2.984	.634
13	.82	.875	.883	.697
14	.62	.755	1.025	.580
15	.29	.543	1.870	.636
16	.29	.586	2.158	.640
17	.43	.640	1.358	.601
18	.75	.689	.508	.665
19	.06	.270	5.140	.556
20	.22	.523	2.631	.494
21	.47	.655	1.319	.757
22	.09	.349	4.457	.228

#### Principal Component Analysis

The Kaiser-Meyer-Olkin coefficient (KMO) and the Bartlett test for sphericity were used to test for the suitability of the dataset for a PCA. According to Cleff (25), the KMO should be ≥0.5. In the CG, the correlation matrix of the 27 items was found to be suitable (KMO = 0.948, Bartlett test for sphericity: p < 0.001). Loadings ranged from 0.176 (item 12) to 0.797 (item 4). According to Krohne and Hock (26), the loadings of the items on a principle component matrix should be >0.3. Except for the items 12 (0.176) and 27 (0.258), all items reached this minimum value.

In the TS group, the correlation matrix of the 27 items was also suitable for PCA (KMO = 0.920, Bartlett test for sphericity: p < 0.001). Loadings ranged from 0.275 (Item 12) to 0.813 (Item 19).

#### Final Version of the RAQ-R

Based on the results of the above-mentioned statistical analyses in the CG, elimination of the following items could be taken into consideration: 3, 6, 11, 12, 14, 16, 17, 20, 24, 26, and 27, while in the TS group, this was the case for items 3, 7, 12, 13, 16, 17, 26, and 27. Since the RAQ-R is supposed to be used in both healthy individuals as well as different psychiatric populations (including TS), we decided to eliminate only items, which came up in both groups (items 3, 12, 16, 17, 26, and 27). Despite being selected for elimination for statistical reasons, item 27 was deemed clinically relevant and therefore was kept.

In summary, following above considerations, the following five items have been deleted from the RAQ-R:

- Item 3: I have rage attacks that make me ashamed.
- Item 12: Because of a rage attack, I am in conflict with the law.
- Item 16: I have thought of going to see a doctor/therapist because of my rage attacks.
- Item 17: When I get angry, it can happen that I become palpable with other people.
- Item 26: I also have age attacks against unfamiliar people.

Accordingly, the RAQ-R was shortened from the initial 27 items to a 22-items version. All subsequent evaluations of the RAQ-R were done exclusively with this final, shortened, 22-items version of the RAQ-R (range, 0–66). The final version of the RAQ-R can be found in the **Supplementary Material**.

### Validation of RAQ-R in the Control Group

#### Convergent and Discriminant Validity

As mentioned before, validity was tested primarily in the CG to avoid distortions. Only for the disease specific assessments (ATQ, GTS-QoL) data from the TS group was used. All correlations were statistically significant, besides the subscale “risk taking” of the I-8. For details see **Table 3**. However, all correlated scales showed relatively low correlation coefficients. The highest correlation was found with the ISR total score (r = 0.43). These low to moderate correlations of comorbid scales were used to establish the discriminant validity, showing that RAQ-R measured a distinct construct, which was not closely related to the one measured by the other scales. The correlations of the RAQ-R with the impulsivity scales BIS-15 and I-8 were all in the low range, with the highest correlation being I-8 “urgency” (r = 0.31). These correlations were meant to establish the convergent validity and showing higher correlations with the construct of impulsivity. Surprisingly, the correlations we found were only low, which indicated that impulsivity is quite distinct from rage attacks.

**Table 3:** Correlations between the RAQ-R and other instruments.

Assessment	Control group		TS group	
	Correlation with RAQ-R	p	Correlation with RAQ-R	p
BIS-15	0.128	0.001	0.296	0.001
I-8				
Urgency	0.310	<0.001	0.461	<0.001
Intention	−0.130	0.001	−0.139	0.119
Endurance	−0.193	<0.001	−0.272	0.002
Risk taking	0.044	0.264	0.028	0.752
ADHD SB	0.353	<0.001	0.450	<0.001
ISR				
Depressive syndrome	0.367	<0.001	0.356	<0.001
Anxiety syndrome	0.258	<0.001	0.228	0.010
OCS	0.318	<0.001	0.303	0.001
Somatoform syndrome	0.213	<0.001	0.116	0.19
Eating disorder syndrome	0.211	<0.001	−0.136	0.129
Supplementary item	0.363	<0.001	0.300	0.010
Total score	0.432	<0.001	0.300	0.001
OCI-R	0.348	<0.001	0.356	<0.001
ATQ*	–	–	0.194	0.029
GTS-QoL*	–	–	0.478	<0.001
QoL-VAS	−0.225	<0.001	−0.296	0.001

*Only in TS group, BIS-15 = Barratt Impulsivness Scale—Short Version, I-8 = Impulsive behavior scale-8, ADHD = attention deficit/hyperactivity disorder, ADHD-SB = ADHD self-assessment scale, ISR = ICD-10 Symptom Rating, OCI-R = Obsessive-Compulsive Inventory-Revised, OCS = obsessive-compulsive syndrome, ATQ = Adult Tic Questionnaire, GTS-QoL = Gilles de la Tourette Syndrome—Quality of Life Scale, QoL-VAS = visual analogue scale of the GTS-QoL.

#### Reliability

In the control group a Cronbach α of 0.939 was calculated.

#### Factor Structure of the RAQ-R

**Figure 1:**
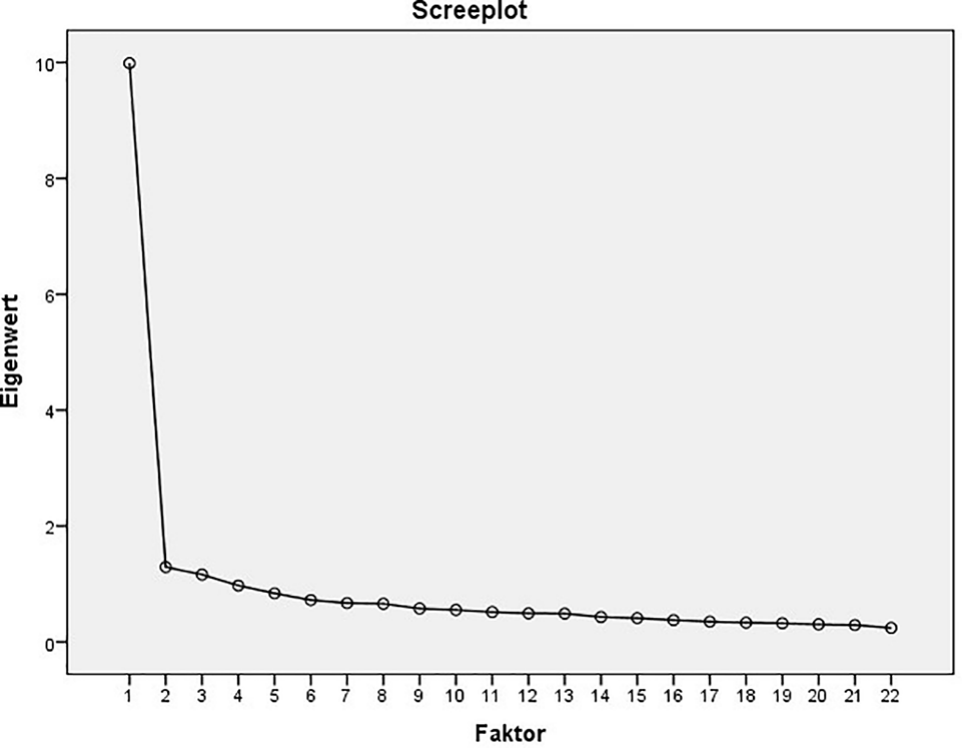
Screenplot of the principal component analysis (PCA) of the 22-items version of the RAQ (data obtained from the control group, N = 645).

The factor structure of the 22-items version of the RAQ-R was examined using a second PCA in the control group. The KMO coefficient (0.957) and the Bartlett test for sphericity (p < 0.001) demonstrate that the data was suitable for a PCA. To determine the number of extracted factors the screeplot was examined (compare **Figure 1**), which clearly indicates a 1-factor structure (27). Therefore, further factor rotation was not necessary. The total amount of explained variance was 45.389%. Loadings ranged from 0.250 to 0.798. For further results of the PCA see **Table 4**.

**Table 4:** Loading of each item of the 22-items version of the RAQ-R.

RAQ-R: Item	Loadings Component 1
1	.701
2	.634
3	.798
4	.686
5	.667
6	.746
7	.771
8	.704
9	.562
10	.717
11	.712
12	.689
13	.734
14	.617
15	.675
16	.682
17	.637
18	.699
19	.612
20	.533
21	.791
22	.250

#### Group Comparisons

Rage attacks as assessed by the RAQ-R (range, 0–66) were significantly more common in patients with TS compared to controls: TS: mean = 25 (sd = 15.35), range, 0–61, CG: mean = 10.37 (sd = 9.48), range, 0–52, p < 0.001 with d *=* 1.37 (1.26; 1.48), indicating a large effect size. In both groups, there were no significant difference in mean RAQ-R scores between male and female [TS: male = 25.51, female = 23.82 (p = 0.572); CG: male = 10.77, female = 10.26 (p = 0.580)], between age groups (TS: p = 0.577; CG: p = 0.267) and between education levels (TS: p = 0.67, CG: p = 0.37). We also found no differences in RAQ-R scores between medicated and unmedicated patients with TS (medicated: mean RAQ-R = 25.5, n = 60, unmedicated: mean RAQ-R = 24.55, n = 67, p = 0.73). In the CG, medicated individuals (n = 15) had a significantly higher mean RAQ-R score (16.07) as compared to unmedicated individuals (10.23, n = 630, p = 0.018).

#### TS Group: Correlations With Tic Severity and Comorbidities

**Table 3** displays correlations between the RAQ-R and further self-assessments in the TS group. The highest correlations were found between the RAQ-R and the GTS-QoL (r = 0.49) and the ADHD-SB (r = 0.45). The correlations between the RAQ-R and the ISR total score (r = 0.30) and all subscales as well as the ATQ (r = 0.19) were low to moderate.

#### Influence of ADHD on Rage Attacks

In both samples, individuals with ADHD (according to ADHD-SB, TS+ADHD) demonstrated significantly higher mean values of the RAQ-R compared to those without ADHD (TS-ADHD) [TS: mean RAQ-R = 18.0 (sd = 14.48) vs. 27.7 (sd = 14.90) (p = 0.001 with d = 0.66, indicating a medium to large effect size), CG: mean RAQ-R = 8.8 (sd = 8.43) vs. 14.2 (sd = 10.75) (p < 0.001, d = 0.59, indicating a medium effect size)].

## Discussion

This study aimed to develop and validate an instrument that can be used to assess rage attacks in adults with TS. This was motivated by the fact that rage attacks have been identified not only as a common symptom in patients with TS, but also a phenomenon that often influences patients’ and families’ quality of life and therefore may cause significant social impairment (2, 3). However, so far no validated instrument is available to measure specifically rage attacks in adult populations with TS. We included not only a large sample of healthy controls, but also a large group of adult patients with TS to test the quality criteria of this newly developed RAQ-R. In addition, we were interested in investigating, whether rage attacks are more common in adults with TS compared to a control group. Since available data are conflicting (2, 9–13, 28), we also wanted to investigate whether rage attacks are associated with other clinical symptoms such as tics and psychiatric comorbidities including ADHD, impulsivity, and OCD.

In line with available literature, we were able to demonstrate that rage attacks are a clinically relevant symptom that occurs not only in children, but also in adults with TS. Rage attacks were found to be more common in patients with TS compared to healthy controls. Although rage attacks occurred more common in individuals with comorbid ADHD, we found only a moderate correlation between rage attacks (as assessed by RAQ-R) and ADHD (as assessed by ADHD-SB). Contrary to our hypothesis, correlations between RAQ-R and the impulsivity scales BIS-15 and I-8 were low. Therefore, it can be assumed that impulsivity (as assessed by I-8 and BIS-15) has to be differentiated from rage attacks (as assessed by the RAQ-R). Interestingly and in contrast to recent studies in children (10), we found only low correlations between obsessive compulsive symptoms (as assess by OCI-R) and tic severity (as assessed by ATQ) and rage attacks. Because of the reported high correlation between the tic self-assessment ATQ and the Yale Global tic Severity Scale (YGTSS) (20), the gold standard for tic assessment, this result can be regarded as reliable.

In line with a small number of recent studies (28), but in contrast to the majority of other studies mainly performed in children (2, 9–13), from our data it is suggested that rage attacks also occur in patients with “TS only”—completely independent from psychiatric comorbidities such as ADHD, impulsivity, and OCD and therefore may represent a discrete comorbidity.

Based on recent studies (29, 30), it is well known that quality of life is reduced in adult patients with TS mainly due to comorbid depression and OCD. Our data corroborates the finding of reduced quality of life, but in addition, demonstrates that rage attacks (as assessed by the RAQ-R) also have a significant negative influence on quality of life in adults with TS. Accordingly, treating physicians should be aware of this symptom, since it may result in significant social impairment.

The quality criteria of the RAQ-R were good to excellent: construct validity demonstrated a 1-factor structure, internal consistency, and discriminant validity were excellent. Since so far validated assessments to measure rage attacks in adults were not available, we decided to use the impulsivity scales BIS-15 and I-8 to test for convergent validity of the RAQ-R. However, correlations were only low and therefore convergent validity could not be reliably investigated.

This study was performed as an online survey. This allowed not only to include a large number of patients with TS and healthy controls within a short time period, but also to recruit a mixed and more representative patient population suffering from mild to severe tics (20). Due to the study design, participants had to provide complete answers and therefore no data were missing. Participation was anonymous, which resulted in our opinion in more truthful statements. Participants received no compensation. The RAQ-R is a standardized and objective instrument, since it includes only closed-ended questions.

The following limitations have to be addressed: (i) due to the study design, only self-assessments and no examiner assessments could be used; (ii) the diagnosis of TS and other tic disorders only based on patients’ judgement. However, we believe that this did not influence our results, since (a) the questionnaire was online only for about two months, (b) only few patients had been informed about the study *via* our TS clinic and advocacy groups, and (c) participants received no compensation. Therefore we believe that patients’ judgement is reliable; (iii) due to the study design, diagnoses of psychiatric comorbidities only based on self-assessments. However, kind, number, and severity of psychiatric comorbidities in our sample were similar to other large samples (3, 31) and therefore can be regarded as representative; (iv) participants of the control group were not personally investigated to exclude psychiatric diseases. However, only a very small number of participants reported psychiatric diagnoses and less than half of these individuals indicated that these symptoms were clinically relevant at time of study participation. Thus, we believe that control can indeed be regarded as *healthy* controls; (v) the education level in our control group was higher than in the TS group. However, we found no association between rage attacks (as assessed by RAQ-R) and education levels. The education in our TS group was similar to previous studies in TS (32, 33). Therefore, we believe that this difference had no influence on our results; (vi) our control group comprised much more female than male, while in the TS group more male were included. This is not surprising, because TS is three to four times more common in male. The high proportion of female in the control group can be best explained by the fact that there are more female than male among both employees and students at MHH; (vii) we decided to use measurements for impulsivity for the assessment of convergent validity instead of measurements for anger. Based on clinical experience, we believe that impulsivity is closer related to rage attacks than anger. In order not to further increase the burden to participants, we therefore decided not to include additional measurements for anger; (viii) unexpectedly the correlations between the RAQ and measures of impulsivity turned out relatively low, which showed that the constructs of impulsivity and rage attacks are quite distinct. That left us with the lack of a closer construct for convergent validity, which is a limitation in our validation process. Further research is needed to further validate the RAQ in other terms of validity, such as criterion validity; (ix) we found a large difference between the TS and the control group regarding rage attacks (assessed by RAQ-R), but we did not test measurement invariance. Therefore, it is possible that both groups interpret the RAQ-R or “rage attacks” in a conceptually different way. In subsequent studies, a new, sufficiently large sample should be used to (a) confirm the factor structure in a confirmatory factor analysis (CFA) and (b) to compare TS patients and other groups to clarify whether rage attacks assessed with the RAQ-R are understood and experienced in the same (or different) way.

In summary, we developed a new instrument to assess rage attacks in adults with TS. This new assessment demonstrates good to excellent quality criteria, however convergent validity could not be assessed. Using the RAQ-R, we found that rage attacks are more common in adults with TS compared to healthy controls. In contrast to recent studies, from our data it is suggested that rage attacks represent a discrete comorbidity in TS that also occurs in patients with “TS only” independently from comorbid ADHD and OCD. Rage attacks as assessed by the RAQ-R can be clearly differentiated from impulsivity. Clinicians should be aware of rage attacks in adults with TS, since they may influence patients’ quality of life significantly.

## Data Availability Statement

The data that support the findings of this study are available from the corresponding author, KMV, upon reasonable request.

## Ethics Statement

The studies involving human participants were reviewed and approved by the Ethics Committee at Hannover Medical School. The patients/participants provided their written informed consent to participate in this study.

## Author Contributions

KM-V, LK, and AP contributed to the conception and design of the study. KM-V, LK, MH, NP, and EJ contributed to the acquisition of data and organized the database. KM-V wrote the first draft of the manuscript. KM-V, LK, MH, LP, and EJ contributed to the analysis and interpretation of data. All authors contributed to manuscript revision, read and approved the submitted version.

## Funding

KM-V has received financial or material research support from the EU (FP7-HEALTH-2011 No. 278367, FP7-PEOPLE-2012-ITN No. 316978), the German Research Foundation (DFG: GZ MU 1527/3-1), the German Ministry of Education and Research (BMBF: 01KG1421), the National Institute of Mental Health (NIMH), the Tourette Gesellschaft Deutschland e.V., the Else-Kroner-Fresenius-Stiftung, and GW, Almirall, Abide Therapeutics, and Therapix Biosiences; has received consultant’s honoraria from Abide Therapeutics, Tilray, Resalo Vertrieb GmbH, and Wayland Group, speaker’s fees from Tilray and Cogitando GmbH.

## Conflict of Interest

The authors declare that the research was conducted in the absence of any commercial or financial relationships that could be construed as a potential conflict of interest.
