# Supplementary material for: The Rage Attack Questionnaire-Revised (RAQ-R): Assessing Rage Attacks in Adults With Tourette Syndrome
Source: Front Psychiatry. 2020 Jan 28;10:956. doi: 10.3389/fpsyt.2019.00956 (PMC6997809; doi:10.3389/fpsyt.2019.00956)
Supplement: Supplementary file 1 [file DataSheet_1.docx]

Rage Attack Questionnaire-Revised version (RAQ-R): English Version

The following list shows some behaviors that people sometimes show. Please read the questions carefully and decide, how much or how little the following statements apply to you. Please tick for each question the number that best suits your assessment. Please, follow your first impulse.

Please refer to your assessment for the last 2 weeks until today.

|  | not at all  never | a little  sometimes | strong  frequent | very strong  very common |
| --- | --- | --- | --- | --- |
| 1. I tend to rage attacks. |  |  |  |  |
| 1. When I get angry, I behave very differently than usual. |  |  |  |  |
| 1. Very different occasions can cause me rage attacks. |  |  |  |  |
| 1. I am afraid of the appearance of my own rage attacks. |  |  |  |  |
| 1. I'm more upset than I want to be. |  |  |  |  |
| 1. It's not nice to witness my rage attacks. |  |  |  |  |
| 1. I can get angry very quickly. ("From 0 to 180") |  |  |  |  |
| 1. When I'm angry, it can happen that I abuse and insult others. |  |  |  |  |
| 1. I feel impaired by my rage attacks. |  |  |  |  |
| 1. Because of an outburst of anger, I have come into conflict with the law. |  |  |  |  |
| 1. I can become disproportionately angry. |  |  |  |  |
| 1. I have problems with other people because of my rage attacks. |  |  |  |  |
| 1. I regret my rage attacks in retrospect. |  |  |  |  |
| 1. In situations where I had a rage attack, I am also tense afterward. |  |  |  |  |
| 1. When I get angry, I lose control of myself. |  |  |  |  |
| 1. When I get angry, it can happen suddenly and unexpectedly. |  |  |  |  |
| 1. Once I get angry, I can hardly calm down. |  |  |  |  |
| 1. I sometimes react more annoyingly than is appropriate in the situation. |  |  |  |  |
| 1. I should get treated for my rage attacks. |  |  |  |  |
| 1. When I am angry, it can happen that I destroy objects. |  |  |  |  |
| 1. I have rage attacks, for which I should apologize. |  |  |  |  |
| 1. When I get angry, it can happen that I hit or hurt myself. |  |  |  |  |

Fragebogen zu Wutausbrüchen: RAQ-R (German Version)

In der folgenden Liste sind einige Verhaltensweisen aufgeführt, die Menschen manchmal zeigen. Bitte lesen Sie die Fragen sorgfältig durch und entscheiden Sie, wie sehr oder wie wenig die nachfolgenden Aussagen auf Sie zutreffen. Bitte kreuzen Sie für jede Frage die Zahl an, die Ihrer Einschätzung am besten entspricht. Folgen Sie dabei Ihrem ersten Impuls.

Bitte beziehen Sie sich bei Ihrer Einschätzung auf die letzten 2 Wochen bis heute.

|  | überhaupt nicht, nie | ein wenig,  manchmal | stark, häufig | sehr stark, sehr häufig |
| --- | --- | --- | --- | --- |
| 1. Ich neige zu Wutausbrüchen. |  |  |  |  |
| 1. Wenn ich wütend bin, verhalte ich mich ganz anders als sonst. |  |  |  |  |
| 1. Ich wünschte, ich könnte meine Wutausbrüche besser kontrollieren. |  |  |  |  |
| 1. Ganz unterschiedliche Anlässe können bei mir Wutausbrüche hervorrufen. |  |  |  |  |
| 1. Ich habe Angst vor dem Auftreten meiner eigenen Wutausbrüche. |  |  |  |  |
| 1. Ich bin aufbrausender, als ich es sein möchte. |  |  |  |  |
| 1. Es ist nicht schön, meine Wutausbrüche mitzuerleben. |  |  |  |  |
| 1. Ich kann sehr schnell wütend werden. („von 0 auf 180“) |  |  |  |  |
| 1. Wenn ich wütend bin, kann es vorkommen, dass ich andere beschimpfe und beleidige. |  |  |  |  |
| 1. Ich fühle mich durch meine Wutausbrüche beeinträchtigt. |  |  |  |  |
| 1. Ich kann unverhältnismäßig wütend werden. |  |  |  |  |
| 1. Ich habe wegen meiner Wutausbrüche Probleme mit anderen Menschen. |  |  |  |  |
| 1. Ich bereue meine Wutausbrüche im Nachhinein. |  |  |  |  |
| 1. In Situationen, in denen ich einen Wutausbruch hatte, bin ich auch danach angespannt. |  |  |  |  |
| 1. Wenn ich wütend bin, verliere ich die Kontrolle über mich. |  |  |  |  |
| 1. Wenn ich wütend werde, kann dies ganz plötzlich und unerwartet auftreten. |  |  |  |  |
| 1. Wenn ich erst einmal wütend bin, kann ich mich kaum beruhigen. |  |  |  |  |
| 1. Ich reagiere zuweilen ärgerlicher, als es in der Situation angemessen ist. |  |  |  |  |
| 1. Ich sollte mich wegen meiner Wutausbrüche behandeln lassen. |  |  |  |  |
| 1. Wenn ich wütend bin, kann es vorkommen, dass ich Gegenstände zerstöre. |  |  |  |  |
| 1. Ich habe Wutausbrüche, für die ich mich entschuldigen sollte. |  |  |  |  |
| 1. Wenn ich wütend bin, kann es vorkommen, dass ich mich selbst schlage oder verletze. |  |  |  |  |
